# COVID-19 Vaccine Booster Dose Fails to Enhance Antibody Response to Omicron Variant in Reinfected Healthcare Workers

**DOI:** 10.3390/v17010078

**Published:** 2025-01-09

**Authors:** Leire Fernández-Ciriza, Álvaro González, José Luis del Pozo, Alejandro Fernandez-Montero, Francisco Carmona-Torre, Paula Martínez de Aguirre, María del Mar Sarasa, Silvia Carlos, Gabriel Reina

**Affiliations:** 1 Department of Microbiology, Clínica Universidad de Navarra, 31008 Pamplona, Spain; lfciriza@unav.es (L.F.-C.); pmartinezdea@unav.es (P.M.d.A.); msarasam@unav.es (M.d.M.S.); gabi@unav.es (G.R.); 2 Department of Biochemistry, Clínica Universidad de Navarra, 31008 Pamplona, Spain; agonzaleh@unav.es; 3 Navarra Institute for Health Research (IdiSNA), 31008 Pamplona, Spain; afmontero@unav.es (A.F.-M.); fcdelatorre@unav.es (F.C.-T.); scarlos@unav.es (S.C.); 4 Infectious Diseases Division, Clínica Universidad de Navarra, 31008 Pamplona, Spain; 5 Department of Occupational Medicine, Universidad de Navarra, 31008 Pamplona, Spain; 6 Department of Preventive Medicine and Public Health, Universidad de Navarra, 31008 Pamplona, Spain

**Keywords:** booster, COVID-19, immunogenicity, mRNA vaccine, Omicron, reinfection

## Abstract

The emergence of new variants and diverse vaccination regimens have raised uncertainty about vaccine effectiveness against SARS-CoV-2. This study aims to investigate the impact of Omicron primo-/reinfection and primary vaccination schedules on the immunogenicity of an mRNA-based booster dose over a six-month period. We conducted a prospective cohort study to assess the durability and level of antibodies of 678 healthcare workers fully vaccinated against COVID-19. They were categorized based on their primary vaccination regimen. Blood samples were collected before the booster dose and 1 and 6 months after. Significant Anti-S-RBD differences were found between previously infected and naïve volunteers (*p* = 0.01). Considering the initial vaccine schedules, mRNA-based vaccines displayed significant higher antibody production and longer persistence among both infected and naïve participants. After the booster dose, participants primoinfected with the Omicron variant exhibited higher antibody concentrations than those who experienced reinfection, even after 6 months of follow-up (22,545 and 9460 U/mL, respectively). Moreover, these groups showed the most pronounced disparity in antibody titers ratios between infected and uninfected individuals. Overall, the booster dose failed to enhance humoral response in individuals reinfected with the Omicron variant after receiving it. Hybrid immunity and mRNA-based vaccine initial schedules showed higher levels and longer persistence of antibodies.

## 1. Introduction

By August 2024, 73.51% of the European Union population had received a complete SARS-CoV-2 vaccination schedule. In Spain, the latest data obtained in May 2023 reveal that the percentage of the population fully vaccinated was 85.18% [1].

Several studies have demonstrated that available adenovirus vector and mRNA vaccines provide significant protection against SARS-CoV-2 by producing antibodies and T cells mediated protection [2]. However, the decrease in antibody levels and, consequently, in vaccine protection over time appears to be real for all forms of infections starting from the sixth month after the second injection [3].

Since the Omicron variant appeared at the end of November 2021, it was proclaimed a variant of concern (VOC) by the WHO due to its higher transmission rate [4]. It is known that this ability is related to its heavy mutations in the spike (S) protein, leading to increased infectivity and antibody evasion [4,5]. In Spain, it was the predominant variant which emerged during week 51 of the year 2021. During the initial weeks of 2022, the BA.1 lineage was predominantly detected (weeks 1–8), subsequently being supplanted by BA.2 (weeks 9–23) [6,7].

Due to the decline in vaccine protection over time and the emergence of variants capable of evading vaccine protection, the number of SARS-CoV-2 infections and reinfections have increased [6]. To curb the rising rates of infections, a campaign to administer a third dose of vaccine against SARS-CoV-2 started in November 2021 [8]. There are data on which long-term monitoring has already been conducted following the booster dose administration, such as the study conducted by Alsoussi et al., in which samples were collected up to 26 weeks (six and a half months) after the booster dose inoculation to study its effect [9]. Other studies have analyzed different vaccination regimens and the effect of booster dose, both homologous and heterologous, reaching the conclusion that heterologous combinations of adenoviral and mRNA vaccines showed slightly different efficacy against the Omicron variant compared to combinations using only mRNA vaccines and also exhibited slower IgG responses compared to those primed with homologous mRNA regimens [10]. Most of these studies have focused on comparisons between mRNA-based vaccine regimens or the heterologous ChAdOx1/BNT162b2 regimen. However, there is a notable lack of data comparing adenoviral vector-based primary regimens (such as ChAdOx1/ChAdOx1 or a single dose of ChAdOx1) with both heterologous and homologous mRNA-based primary regimens (e.g., mRNA-1273/mRNA-1273, BNT162b2/BNT162b2, or ChAdOx1/BNT162b2). This gap persists despite the well-established understanding that adenoviral vector-based vaccines typically generate lower antibody levels, as previously described [10,11]. Further data on different homologous and heterologous combinations of mRNA or adenovirus-based vaccines with an mRNA booster dose are needed to compare the immunogenicity of each vaccine simultaneously and during a longer follow-up [12].

To understand the effects of different factors on vaccination effectiveness, we conducted this study with the aim of analyzing the immunogenicity induced by the booster dose in populations that were either primoinfected or reinfected with the Omicron variant after six months of follow-up. Additionally, we examined the immunogenic effect of primary vaccination schedules on antibody production after booster dose.

## 2. Materials and Methods

### 2.1. Study Design and Participants

A prospective cohort study started in March 2021 to assess durability and level of humoral response (SARS-CoV-2 Vaccine Immunity Navarra—SAVIN Project). All healthcare workers at Clinica Universidad de Navarra (Pamplona, Spain) who had received full coverage (two doses) of COVID-19 vaccines were invited to participate voluntarily. The actual analysis was carried out between December 2021 and June 2022 (six months after COVID-19 vaccine booster dose).

**Figure 1:**
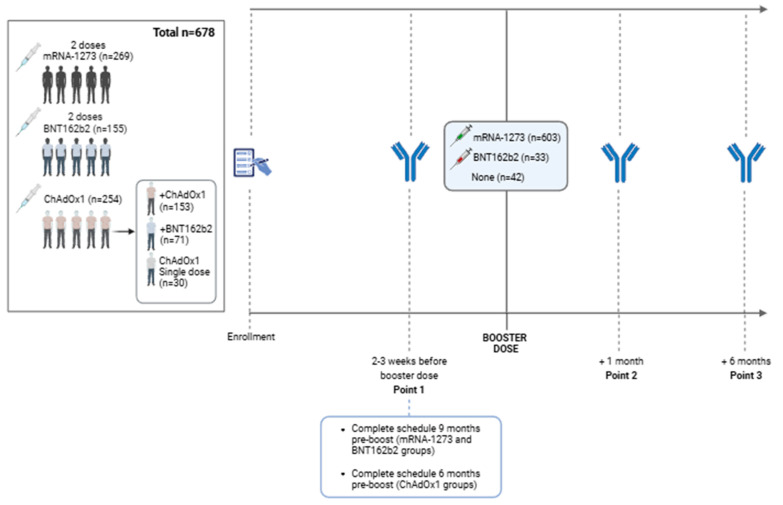
Flowchart of SAVIN participants. Recruitment and follow-up summary for humoral response evaluation before and after booster dose (created in BioRender.com).

Six hundred and seventy-eight subjects were enrolled in the study (Table S1) (out of 709 individuals initially enrolled in the SAVIN study [13]), and they were classified depending on their primary vaccination schedule (group 1: mRNA-1273 vaccine; group 2: BNT162b2 vaccine; groups 3/4/5: ChAdOx1 nCoV-19 as first dose vaccine). For mRNA-1273 and BNT162b2, the vaccination schedule included 2 doses for all participants (previously infected and noninfected individuals) separated by 28 and 21 days, respectively. For those who received a first dose of the ChAdOx1 vaccine and had not been previously infected, they received a second dose of ChAdOx1 (group 3) or BNT162b2 (group 4) vaccine, depending on their choice, 12 weeks after the first shot. Individuals previously infected and vaccinated with a first dose of the ChAdOx1 vaccine did not receive any second dose (group 5) (Figure 1). Enrolment details and the proportion of participants within this cohort can be found elsewhere [14].

Within the 678 fully vaccinated participants, the third dose (booster) was delivered to 603 individuals with the mRNA-1273 vaccine and 33 with the BNT162b2 vaccine; 42 did not receive any booster. Plasma samples were collected from all participants (n = 678) with the aim of studying the humoral immunity generated at three different time points: prior to the booster dose (point 1) and one month (point 2) and six months (point 3) after its vaccination (Figure 1). Additionally, antibodies induced by natural infection were also analyzed at the same time points. Participants’ data were recorded from the hospital register.

In the primary vaccination schedule, one dose of the BNT162b2 vaccine had 30 micrograms of mRNA, while one dose of mRNA-1273 had 100 micrograms. One dose of ChAdOx1 vaccine was composed of no fewer than 2.5 × 10^8^ adenovirus infectious units. For the booster doses, the BNT162b2 vaccine was kept at the same dose, while the mRNA-1273 had a reduced dose (50 micrograms of mRNA) [14,15,16,17].

### 2.2. Humoral Response Evaluation

Anti-SARS-CoV-2 antibody detection was performed using two different commercial chemiluminescence tests. Quantitative detection of total antibodies (IgG and IgM) against the receptor-binding domain (RBD) of the SARS-CoV-2 spike protein (S) and qualitative detection of total antibodies (IgG and IgM) against viral nucleocapsid (Anti-N) were performed using Elecsys^®^ reagents (Anti-SARS-CoV-2-S and Anti-SARS-CoV-2 test. Roche Diagnostics, Germany). Anti-S-RBD levels were quantified (humoral response induced by vaccines and SARS-CoV-2 infection) and considered reactive or non-reactive using a cut-off level of 0.8 U/mL, and levels were quantified up to 200,000 U/mL. Anti-N was interpreted as reactive (previous natural infection) or non-reactive using a cutoff index (COI) of 0.150, as previously proposed, as this is a follow-up study [13]. Anti-N official cutoff index (COI) supplied by the manufacturer is 1.0. Primary infections and reinfections were recorded considering the anti-N data obtained in samples collected over 7 monitoring points between March 2021 and June 2022. No additional antigenic or molecular tests were performed to define previous infections. In the same way, the variant responsible for COVID-19 infection could be inferred throughout the follow-up.

### 2.3. Statistical Analysis

Due to their non-normal distribution determined with the Shapiro–Wilks tests, concentrations were expressed as median and interquartile range (IQR), and non-parametric Mann–Whitney U test was applied to assess the statistical differences in antibody levels.

To compare changes over time depending on the initial vaccine schedule or infection/reinfection patterns, we calculated ratios of the antibody levels between 1 and 6 months of follow-up (points 2 and 3) and compared them with antibody levels before the booster dose (point 1). Ratios were calculated using medians of each study volunteer and were reported for each vaccination schedule group and time points previously specified. To calculate *p*-values between schedules, the mRNA-1273/mRNA-1273 schedule was used as the reference.

To compare immunogenicity depending on infection/reinfection patterns, we calculated ratios of the antibody levels stratified for each group of study (primoinfected in December 2021; primoinfected or reinfected January 2022, or January–June 2022) compared with the noninfected group [18].

A multiple linear regression analysis of Anti-S-RBD levels was conducted, adjusted for age and sex, to investigate the association between different initial vaccination schedules and various scenarios of primary infection or reinfection.

Statistics and graphs were obtained using Stata 15.0. and GraphPad Prism 5 Software. A two-tailed *p*-value < 0.05 was considered statistically significant.

## 3. Results

### 3.1. Serological Response According to Booster Dose Administered

The participants were grouped according to the booster received. Table 1 shows the antibody levels achieved after 1 and 6 months of follow-up after receiving an mRNA-1273 or BNT162b2 booster or none in previously SARS-CoV-2 infected or noninfected individuals.

Anti-S-RBD levels were significantly higher among volunteers previously infected by SARS-CoV-2 in contrast to those who had not been infected in both booster groups after 1 and 6 months of follow-up (*p* < 0.01). Antibody titers were similar after 1 month of follow-up between boosted and non-boosted individuals with previous COVID-19 infection, likely due to the increase in the percentage of infected individuals (from 26.2 to 78%) at this point of follow-up (Table 1). Significant differences emerged after 6 months of follow-up between the mRNA-1273-boosted group and the non-boosted group (*p* = 0.03); however, no significant differences were found with the BNT162b2 group (*p* = 0.43), probably due to the small sample size (n = 33). Among naïve participants, Anti-S-RBD differences were found between the mRNA-1273 and BNT162b2 booster groups (*p* = 0.01) at the first follow-up, whereas no significant differences were found at the second follow-up (*p* = 0.46). 

### 3.2. Serological Response According to Initial Vaccine Schedule

**Figure 2:**
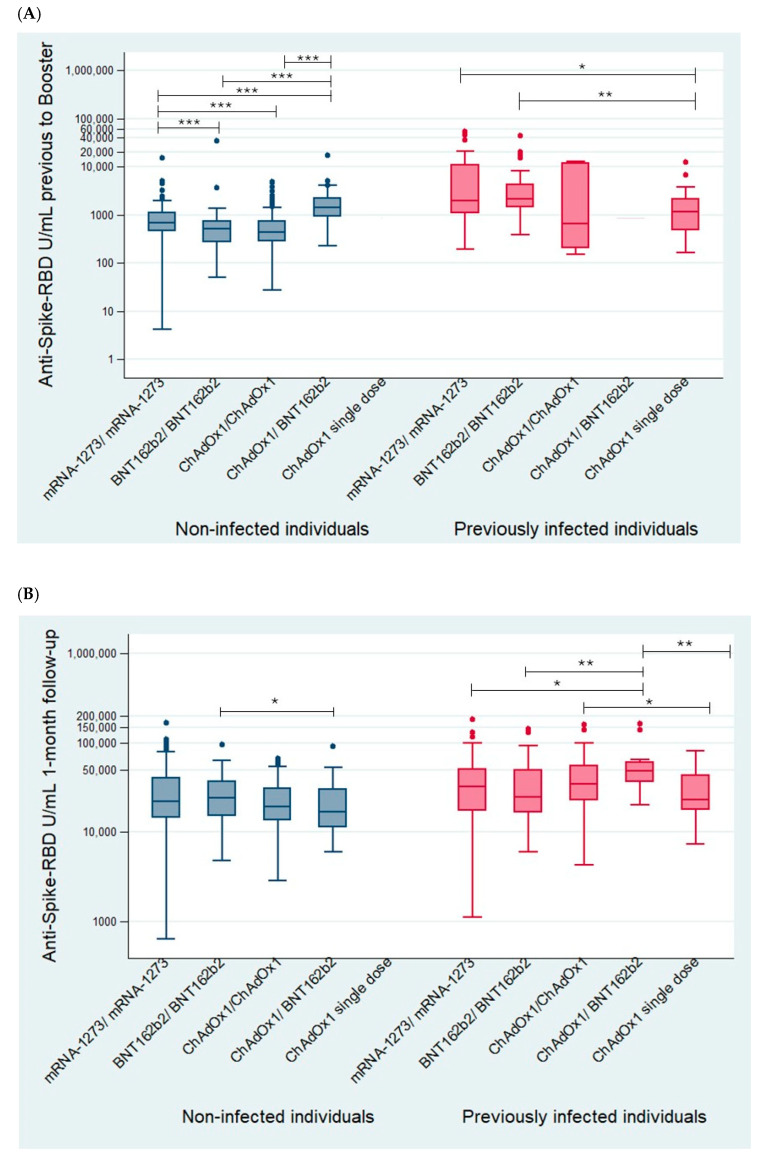

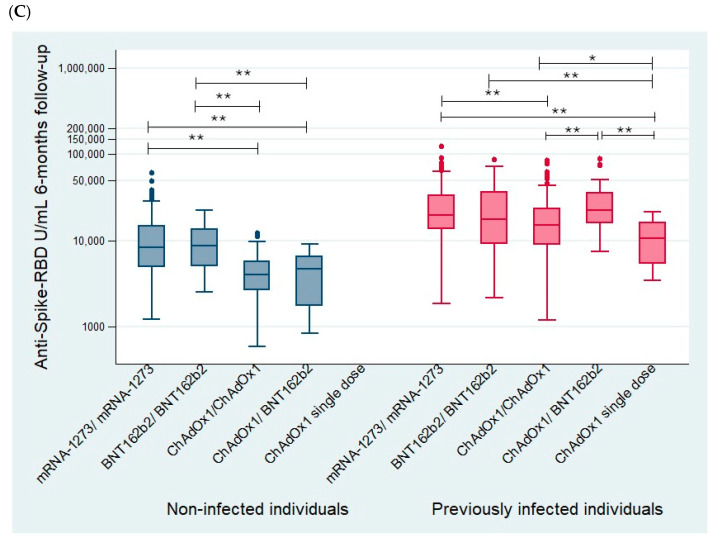
Results of humoral response (Anti-S-RBD total antibody levels (IgG + IgM) (U/mL)) according to the initial vaccine schedule before (**A**), 1 month (**B**), and 6 months (**C**) after booster with mRNA-1273 (n = 603). Statistically significant differences: * (<0.05), ** (<0.01), or *** (<0.001). The Mann–Whitney U test was used to calculate *p*-values.

The Anti-S-RBD antibody levels prior to the booster and induced after mRNA-1273 booster administration were analyzed, considering the initial vaccination schedule (Figure 2, Table S2).

In the group of previously infected volunteers, after 1 month of follow-up (Figure 2B), significantly higher levels of Anti-S-RBD antibodies were found in individuals vaccinated with the ChAdOx1/BNT162b2 schedule compared with other vaccination schedules (mRNA-1273/mRNA-1273 *p* < 0.05; BNT162b2/BNT162b2 and ChAdOx1 single dose *p* < 0.01) except for ChAdOx1/ChAdOx1 (*p* = 0.18). In addition, significant differences were found between ChAdOx1/ChAdOx1 and ChAdOx1 single-dose schedules (*p* < 0.05). No significant differences were found among the remaining vaccination schedules. In the group of previously infected volunteers, after six months of follow-up (Figure 2C), statistically significant differences were observed in Anti-S-RBD antibody levels between the homologous schedules and the heterologous schedules composed of only one mRNA-based vaccine (mRNA-1273/mRNA-1273 vs. ChAdOx1/ChAdOx1 and ChAdOx1 single dose *p* < 0.01; BNT162b2/BNT162b2 vs. ChAdOx1 single dose *p* < 0.01), with the levels of homologous schedules being higher. Heterologous schedules composed of two mRNA vaccines versus one were also compared. Significantly higher levels of antibodies were found in the ChAdOx1/BNT162b2 group regimen (*p* < 0.01) compared with the ChAdOx1/ChAdOx1 or ChAdOx1 single-dose regimens after 6 months of follow-up (Figure 2C). It is worth noting that in the infected group, all regimens were more immunogenic (*p* < 0.01) than the ChAdOx1 single-dose regimen at the last follow-up point despite the booster doses (Figure 2C).

Among individuals in the naïve group after six months of follow-up (Figure 2C), the regimens composed of 3 doses of mRNA vaccines produced significantly more persistent antibodies than the ChAdOx1/BNT162b2 and ChAdOx1/ChAdOx1 regimens (2 and 1 dose of mRNA vaccines, respectively) (*p* < 0.01). The adjusted linear regression analysis confirmed these findings, revealing an association between improved humoral immunogenicity and the mRNA-based vaccines received in the initial 2021 vaccination schedule.

Ratios were calculated to assess the change in antibody levels over time. Regarding 1 month and 6 months/pre-booster ratios, it was observed that in heterologous schedules (including heterologous schedule with different mRNA vaccines: BNT162b2/BNT162b2 plus mRNA-1273 booster dose), the effect was higher than in homologous schedules (mRNA-1273/mRNA-1273 plus mRNA-1273 booster dose), both in previously infected and noninfected individuals, and some of the differences were statistically significant (Table 2). However, the ChAdOx1/ChAdOx1 schedule in previously infected individuals does not reach statistical significance in previously infected individuals, despite its ratio being higher compared to the mRNA-1273/mRNA-1273 schedule (ratio of 18.44 versus 1.60), likely due to the small number of individuals in its group (n = 6).

From the perspective of persistence, the schedules demonstrating the longest duration in both infected and noninfected individuals following the booster are those based on mRNA vaccines (statistically significant difference taking the mRNA-1273/mRNA-1273 schedule as a reference; Table 2).

It is worth noting that the ChAdOx1/BNT162b2 schedule shows significantly low values among noninfected individuals in all calculated ratios, likely associated with the difference in group size compared to the other groups.

The main findings of this analysis were that mRNA-based vaccines induced higher and longer-lasting antibody levels and that previous infections increased the persistence of the antibody response.

### 3.3. Serological Response Depending on Natural Infections or Reinfections

**Figure 3:**
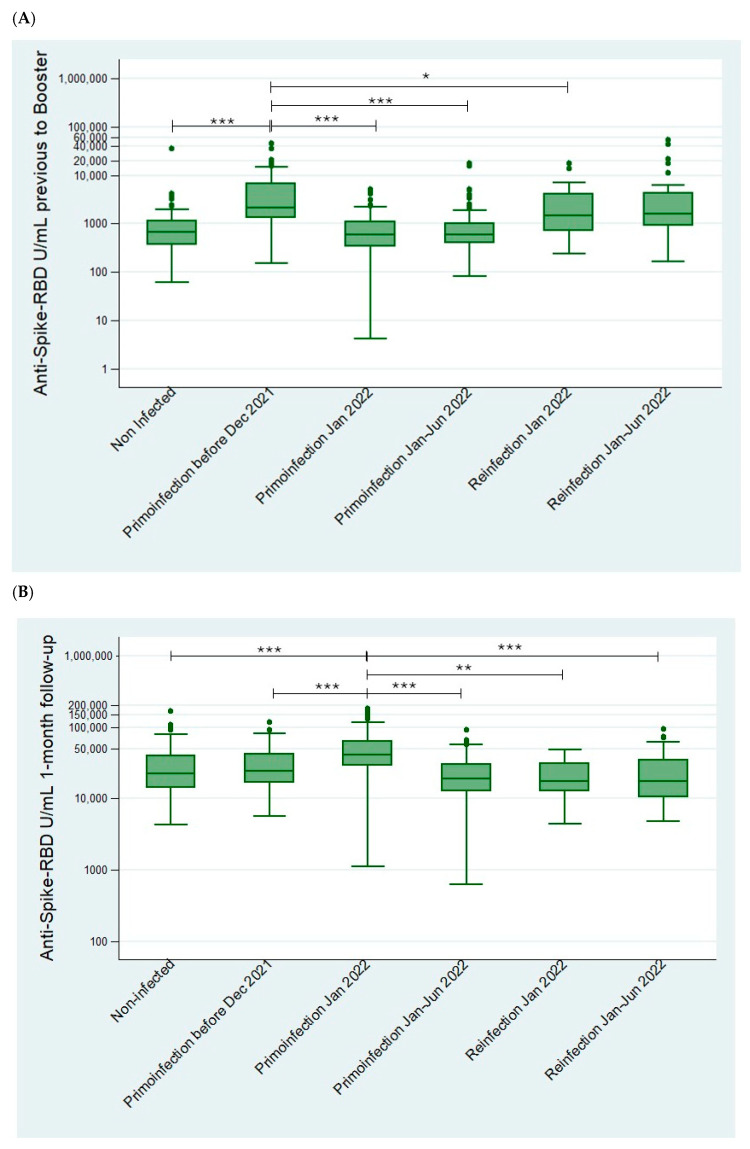

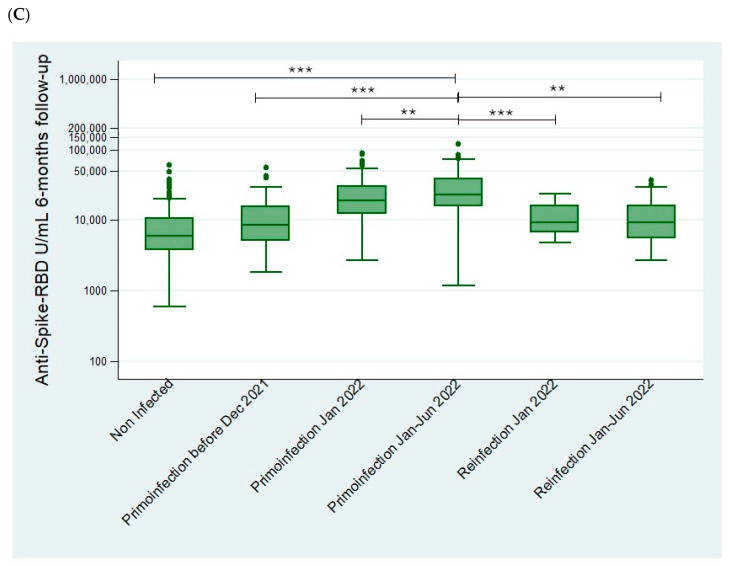
Result of humoral response (Anti-S-RBD total antibody levels (IgG + IgM) (U/mL)) among noninfected and infected individuals (primo- and reinfected) before (**A**), 1 month (**B**) and 6 months (**C**) after booster with mRNA-1273 (n = 603). Statistically significant differences: * (<0.05), ** (<0.01), or *** (<0.001). The Mann–Whitney U test was used to calculate *p*-values.

The hybrid immunity at different follow-up points prior to and after booster administration was also studied. As explained above, primary infections and reinfections were established according to Anti-N index levels, which allowed us to confirm whether an infection or a reinfection has occurred during the follow-up, and Anti-S-RBD titers were studied among the different subgroups (Figure 3, Table S3).

The antibodies obtained in the primoinfection and reinfection groups in January 2022 were compared after one month of follow-up. Antibody levels in the primoinfected group were significantly higher than in the reinfected group (*p* < 0.01) and also exhibited statistically significant differences compared to all of the other groups (Figure 3B). Moreover, when the “Primoinfection before December 2021” group was compared to the Omicron reinfection group in January, the reinfection group also had lower median antibody titers, although not significant (*p* = 0.052). After six months of follow-up, following the same pattern observed at the 1-month follow-up point, the recently primoinfected group (primoinfection in January–June 2022) also exhibited statistically significant differences compared to all other groups (Figure 3C). Failure of the immune-boosting effect after administration of the booster dose was observed in those participants who were reinfected with the Omicron variant. The adjusted linear regression analysis conducted at this stage confirmed that participants reinfected with the Omicron variant in 2022, after receiving the booster dose in December 2021, did not exhibit higher Anti-S-RBD levels compared to those who were not reinfected during this period (*p* = 0.111–0.631). In addition, this analysis did confirm the higher humoral response developed by those primoinfected after the booster dose, compared to both noninfected and reinfected individuals (*p* < 0.001).

When calculating antibody ratios between uninfected and infected volunteers (Table 3), after one month of follow-up volunteers primoinfected in January have the highest ratio. After six months of follow-up, volunteers primoinfected with the Omicron variant have the highest ratio too. This does not occur in reinfected volunteers, nor in those primoinfected with older variants such as wild-type, Alpha, or Delta.

## 4. Discussion

The main finding of this study is the failure of the booster dose to enhance antibody response following Omicron reinfection (firstly primoinfected by ancestral variants). These results are consistent with a study conducted by Reynolds et al., who observed that Omicron infection can boost binding and neutralizing antibody responses against Omicron and other VOCs in triple-vaccinated naïve healthcare workers. However, this phenomenon does not apply to individuals primoinfected with the Wuhan Hu-1 variant [19]. The specific sequence of vaccination and infection during the initial Wuhan Hu-1 wave followed by the Omicron wave introduces a new concept: “hybrid immune damping” [19,20]. This “damping” of booster dose may be due to pre-existing immunological memory, which may limit “de novo” reactivity to non-conserved epitopes (phenomenon of antibody feedback). Consequently, antigen elimination will be stimulated reducing the support to B-cell immune response. In line with this, it has been described that the response against the ancestral variant (wild-type) remains predominant after reinfection with a new variant. This is closely linked to ‘immune imprinting’, as it causes post-vaccination infections (breakthrough infections) with non-ancestral variants to recall cross-reactive memory B cells elicited by wild-type-based vaccines, but rarely produce variant-specific B cells [21,22]. However, this problem may be overcome by increasing antigen dose and/or including adjuvants [23].

Another remarkable finding of the study is that schedules including mRNA-based vaccines produce higher levels of antibodies which persisted longer over time in previously infected and naïve individuals. These findings are in line with a report where ChAdOx1/BNT162b2 and BNT162b2/BNT162b2 schedules followed by BNT162b2 booster dose were compared in naïve people [24]. ChAdOx1/BNT162b2 cohort showed significantly lower binding antibody titers after 6 months of follow-up compared to the BNT162b2/BNT162b2 cohort after being measured by two different methods of commercial chemiluminescent immunoassay (CLIA) technique. Additionally, those authors analyzed the neutralization activity of the antibodies at the same follow-up point, and it was significantly lower in the ChAdOx1/BNT162b2 cohort too [24]. It is noteworthy that, in the cohort of previously infected volunteers, the differences are less substantial between the vaccination schedule groups (no differences were found between 2 and 3 mRNA doses). This result suggests that previous infection could narrow the gap between homologous and heterologous schedules [24]. Regarding the persistence of antibodies depending on primary schedules, the same study reports that priming vaccination composed of 3 mRNA vaccines has the highest persistence in antibody levels, indicating faster declines in the ChAdOx1/BNT162b2 cohort in both binding and neutralizing antibody levels [24]. Similar results in the immunogenicity and durability of antibodies were obtained in a study evaluating three doses of CoronaVac versus two doses of CoronaVac followed by BNT162b2 booster dose [25] and in another comparing a primary regimen of Ad26.COV2.S followed by the same vaccine against boosters based on mRNA technology [26]. In a study conducted by Orlandi et al., the humoral response of individuals vaccinated with either a homologous mRNA schedule (BNT162b6/BNT162b2), a homologous adenovirus-based schedule (ChAdOx1/ChAdOx1), or a heterologous (ChAdOx1/BNT162b2) schedule was analyzed over a six-month period. The results showed that the heterologous ChAdOx1/BNT162b2 vaccination induced a significantly higher antibody response than both the ChAdOx1/ChAdOx1 and BNT162b6/BNT162b2 vaccinations at all follow-up points. However, the differences between ChAdOx1/BNT162b2 and BNT162b6/BNT162b2 vaccinations decreased after four months of follow-up and became statistically non-significant after six months, demonstrating that mRNA vaccines-based schedules produce higher levels of antibodies, which were more persistent. The study also analyzed clinical variables influencing the immune response: only the vaccination schedule had a significant impact on both antibody titers and kinetic parameters, while sex and age were not significant factors [27]. In terms of increased persistence in those previously infected, a study by Panke Qu et al. found that healthcare workers with prior SARS-CoV-2 infection experienced a slower decline in neutralizing antibody titers 7 to 9 months post-booster [28], demonstrating that changes in antibodies in previously infected people are evident over time.

Anti-S-RBD levels were similar after 1 month of follow-up between boosted and non-boosted individuals with previous COVID-19. This was due to the high rate of primary infection within the non-boosted group following the beginning of Omicron circulation. Among those participants without previous SARS-CoV-2 infection, mRNA-1273 booster dose induced higher antibody levels than BNT162b2. This could be explained by the BNT162b2 dose containing 30 micrograms of mRNA in contrast to the 50 micrograms of mRNA present in the mRNA-1273 booster [14,15].

There were several limitations in our study. First, all participants received a booster of mRNA-based vaccines, so no information about immunogenicity of other vaccine technologies could be obtained. Second, past SARS-CoV-2 infections were only determined by serological techniques and the exact moment of infection was unknown; however, a close follow-up based on seven-time-point blood testing over a 15-month period was carried out to estimate the infection time. In addition, there are some limitations inherent to an observational study; we may have a participation bias, as our participants were volunteers who decided to join the cohort within all the hospital staff invited to participate. Moreover, cellular immune response was not considered in this study. Finally, the groups of volunteers in some statistical analyses are not balanced and the sex distribution is unequal, which influences the statistical analyses and the precision of the estimates. On the other hand, the study has several notable strengths. To our knowledge, this is one of the few studies to assess the impact of infections and reinfections with ancestral and/or Omicron variants and their immunogenic effect on the booster dose or the impact of booster doses given previous vaccination schedules. Furthermore, there is a scarcity of studies that directly compare volunteers who have not received booster doses with those who have. In addition, this study makes use of validated robust serological platforms to measure values of interest for the study.

As the attenuating impact of Omicron variant reinfection on vaccine efficacy becomes apparent, further studies are needed to observe vaccine response following reinfections with schedules adapted to new variants. Similarly, given the observed enhanced immunogenicity and prolonged antibody duration associated with hybrid immunity and mRNA-based vaccines, it is advisable to sustain surveillance of booster effects in populations vaccinated with non mRNA-based vaccine schedules.

## Figures and Tables

**Table 1:** Description of participants according to the type of booster vaccine inoculated.

	Booster Vaccine Administered			
	mRNA-1273	BNT162b2	No Booster	TOTAL
N	603	33	42	678
Age, mean (SD)	44.5 (10.8)	52.4 (12.3)	40.7 (10.4)	44.6 (11.0)
Women (%)	86.2	78.8	92.9	86.3
BMI median (IQR)	22.6 (20.7–25.3)	22.8 (20.2–25.7)	21.6 (20.2–25.4)	22.6 (20.6–25.3)
**Anti-S-RBD U/mL median (IQR) in SARS-CoV-2-infected individuals**				
Prior to Booster	1924 (990–5429)	3741 (1463–9975)	9880 (4917–25,837)	2002 (1041–6630)
Booster 1-month follow-up	32,176 (18,024–51,782)	30,499 (14,372–39,649)	31,599 (10,988–50,632)	32,125 (17,461–51,513)
Booster 6-month follow-up	17,686 (10,509–32,045)	16,222 (10,320–20,041)	13,517 (6301–20,642)	17,298 (10,281–31,263)
**Anti-S-RBD U/mL median (IQR) in SARS-CoV-2-noninfected individuals**				
Prior to Booster	633 (358–1094)	320 (210–717)	800 (354–1300)	633 (347–1101)
Booster 1-month follow-up	20,839 (13,489–34,729)	14,495 (6988–21,401)	527 (250–2098)	20,257 (12,770–33,529)
Booster 6-month follow-up	6034 (3778–10,369)	4850 (4426–4893)	- *	5901 (3800–10,110)
**Percentage of SARS-CoV-2 infections (%)**				
Prior to Booster	20.2	30.3	26.2	21.1
Booster 1-month follow-up	42.3	53.1	78.0	45.0
Booster 6-month follow-up	73.9	90.0	100	76.1

* All participants without booster were infected by SARS-CoV-2 by the 6-month follow-up.

**Table 2:** Persistence ratios for analyzed schedules after booster dose with mRNA-1273.

	mRNA-1273/mRNA-1273	BNT162b2/BNT162b2	ChAdOx1/ ChAdOx1	ChAdOx1/BNT162b2	ChAdOx1 Single Dose
**Persistence ratios in SARS-CoV-2-infected individuals**					
1 m/pre-booster ratio	4.28	9.85 *	35.89 *	-	18.46 ***
6 m/pre-booster ratio	1.60	4.44 *	18.44	-	5.92 **
6 m/1 m ratio (between follow-up points)	0.48	0.49	0.33 *	0.38	0.34
**Persistence ratios in SARS-CoV-2-noninfected individuals**					
1 m/pre-booster ratio	30.86	46.10 *	43.00 *	15.30 ***	-
6 m/pre-booster ratio	11.08	15.03	8.84	3.34 ***	-
6 m/1 m ratio (between follow-up points)	0.30	0.31	0.19 ***	0.20 ***	-

1 m: 1-month follow-up; 6 m: 6-month follow-up. Statistically significant differences: * (<0.05), ** (<0.01), or *** (<0.001) (reference for calculating *p*-values was mRNA-1273/mRNA-1273 schedule).

**Table 3:** Ratios for noninfected (reference) and infected individuals at different time points and SARS-CoV-2 variants (only those participants who received mRNA-1273 Booster dose were analyzed (n = 603)).

	Primoinfection Before Dec 2021 vs. Noninfected Ratio	Primoinfection Jan 2022 vs. Noninfected Ratio	Reinfection Jan 2022 vs. Noninfected Ratio	Primoinfection Jan–Jun 2022 vs. Noninfected Ratio	Reinfection Jan–Jun 2022 vs. Noninfected Ratio
**Infecting** **variant**	Wild-type, Alpha, Delta	Omicron (BA.1)	Omicron (BA.1)	Omicron (BA.1, BA.2)	Omicron (BA.1, BA.2)
**Prior to Booster**	3.4	0.93	2.23	0.91	2.70
**Booster** **1-month** **follow-up**	1.08	1.76	0.74	0.82	0.79
**Booster** **6-months** **follow-up**	1.54	3.02	1.56	3.82	1.60

## Data Availability

The data that support the findings of this study are openly available in the Harvard Dataverse at https://doi.org/10.7910/DVN/CYGBZS (accessed on 7 January 2025).

## Supplementary Materials

The following supporting information can be downloaded at: https://www.mdpi.com/article/10.3390/v17010078/s1, Table S1. Distribution of workplaces in healthcare workers; Table S2. Results of humoral response according to the initial vaccine schedule before booster with mRNA-1273 (n = 603); Table S3. Anti-S-RBD levels among noninfected and infected individuals at different time points and SARS-CoV-2 variants (only those participants who received mRNA-1273 Booster dose were analyzed).
