# Supplementary material for: COVID-19 Vaccine Booster Dose Fails to Enhance Antibody Response to Omicron Variant in Reinfected Healthcare Workers
Source: Viruses. 2025 Jan 9;17(1):78. doi: 10.3390/v17010078 (PMC11769551; doi:10.3390/v17010078)
Supplement: Supplementary file 1 [file viruses-17-00078-s001.zip › viruses-3283995-supplementary.pdf]

SUPPLEMENTARY MATERIALS

Table S1. Distribution of workplaces in healthcare workers.

HEALTH CARE WORKER DISTRIBUTION	N	%
**Nursing staff**	293	43.2
**Physicians**	102	15.0
**Laboratory staff**	88	13.0
**Administration or maintenance workers**	185	27.3
**Allied health professionals**	10	1.5
TOTAL	**678**	**100**

Table S2. Results of humoral response according to the initial vaccine schedule before booster with mRNA-1273 (n= 603).

	**mRNA-1273/ mRNA-1273**	**BNT162b2/ BNT162b2**	**ChAdOx1/ ChAdOx1**	**ChAdOx1/ BNT162b2**	**ChAdOx1 single dose**
**N**	256	125	141	58	23
**Anti-S-RBD U/mL median (IQR) SARS-CoV-2 infected individuals**					
**Previous to Booster**	1,962 (1,045-11,491)	2,108 (1,394-4,405)	657 (197-11,817)	-	1,153 (473-2,171)
**Booster 1-month follow-up**	32,517 (17,172-51,347)	24,727 (16,504-49,385)	34,902 (22,506-55,952)	48,537 (34,282-61,356)	23,011 (17,634-43,130)
**Booster 6-months follow-up**	19,616 (13,372-33,476)	17,710 (9,054-36,679)	15,142 (8,865-23,553)	22,474 (15,733-35,647)	10,519 (5,196-16,123)
**Anti-S-RBD U/mL median (IQR) SARS-CoV-2 non-infected individuals**					
**Previous to Booster**	686 (450-1,113)	512 (267-733)	429 (273-736)	1,419 (877-2,201)	-
**Booster 1-month follow-up**	21,903 (14,448-40,227)	23,951 (15,055-37,437)	19,540 (13,489-30,810)	17,071 (11,207-30,199)	-
**Booster 6-months follow-up**	8,295 (4,834-14,736)	8,590 (4,874-13,402)	3,975 (2,632-5,758)	4,679 (1,702-6,768)	-

Table S3. Anti-S-RBD levels among non-infected and infected individuals at different timepoints and SARS-CoV-2 variants (only those participants who received mRNA-1273 Booster dose were analyzed).

	Primoinfection before Dec 2021	Primoinfection Jan 2022	Reinfection Jan 2022	Primoinfection Jan-Jun 2022	Reinfection Jan-Jun 2022	Non Infected	TOTAL
**Infecting Variant**	Wild-type, Alpha, Delta[6,7]	Omicron (BA.1)[6,7]	Omicron (BA.1)[6,7]	Omicron (BA.1, BA.2)[6,7]	Omicron (BA.1, BA.2)[6,7]	-	
**N (%)**	58	134	23	183	38	167	603
**SARS-CoV-2 Anti-S-RBD U/mL median (IQR)**							
**Previous to Booster**	2,261 (1,433-9,999)	620 (340-1,089)	1,484 (669-4,227)	60 (344-1,011)	1,798 (948-5,752)	665 (375-1,131)	732 (401-1,466)
**Booster 1-month follow-up**	24,088 (16,198-42,523)	39,413 (28,536-62,594)	16,693 (12,358-30,120)	18,316 (11,664-30,018)	17,523 (10,490-35,564)	22,336 (13,756-39,686)	23,467 (14,774-41,185)
**Booster 6-months follow-up**	9,111 (4,881-16,714)	17,823 (12,029-28,496)	9,184 (6,558-16,013)	22,545 (15,266-39,222)	9,460 (5,837-16,520)	5,900 (3,778-10,369)	14,147 (7,070-23,882)
**SARS-CoV-2 Anti-N Index median (IQR)**							
**Previous to Booster**	17.1 (4.51-54.08)	0.089 (0.085-0.094)	3.71 (0.794-10.56)	0.089 (0.084-0.095)	18.1 (2.21-42.0)	0.09 (0.084-0.095)	0.092 (0.086-0.106)
**Booster 1-month follow-up**	14.47 (3.31-39.54)	7.29 (2.21-15.58)	145.1 (111.7-190.2)	0.056 (0.051-0.088)	10.9 (1.76-26.2)	0.057 (0.051-0.087)	0.094 (0.055-7.75)
**Booster 6-months follow-up**	13.36 (2.58-28.58)	14.29 (5.47-29.54)	91.39 (51.9-174.6)	15.6 (5.65-35.09)	106 (67.6-170.5)	0.087 (0.084-0.091)	10.2 (0.22-38.4)
